# Novel Bluetongue Virus in Goats, Corsica, France, 2014

**DOI:** 10.3201/eid2012.140924

**Published:** 2014-12

**Authors:** Stéphan Zientara, Corinne Sailleau, Cyril Viarouge, Dirck Höper, Martin Beer, Maria Jenckel, Bernd Hoffmann, Aurore Romey, Labib Bakkali-Kassimi, Aurore Fablet, Damien Vitour, Emmanuel Bréard

**Affiliations:** ANSES (French Agency for Food, Environmental and Occupational Health and Safety);; Maisons-Alfort, France (S. Zientara, C. Sailleau, C. Viarouge, A. Romey, L. Bakkali-Kassimi, A. Fablet, D. Vitour, E. Bréard);; Friedrich-Loeffler-Institut, Greifswald-Insel Riems, Germany (D. Höper, M. Beer, M Jenckel, B. Hoffmann)

**Keywords:** bluetongue virus, novel virus, emergence, Corsica, France, viruses, goats, ruminants

## Abstract

During 2000–2013, 4 genotypes of bluetongue virus (BTV) were detected in Corsica, France. At the end of 2013, a compulsory BTV-1 vaccination campaign was initiated among domestic ruminants; biological samples from goats were tested as part of a corresponding monitoring program. A BTV strain with nucleotide sequences suggestive of a novel serotype was detected.

Bluetongue is an infectious, noncontagious, arthropodborne viral disease of domestic and wild ruminants (*1*). Twenty-six distinct bluetongue virus (BTV) serotypes have been identified (*2*).

The first detection of bluetongue virus (BTV) on the island of Corsica, France, was in 2000, when sheep were found to be infected with BTV genotype 2 (BTV-2); the virus most likely originated from Sardinia (*3*). In 2003 and 2004, BTV-4 and BTV-16 were detected on Corsica (*4*). Since 2004, no other outbreaks of BTV had been reported on the island until September 2, 2013, when BTV-1 was isolated from 3 flocks of sheep in southern Corsica (*5*). In the following weeks, the virus spread across the island, and by March 2014, 169 outbreaks had been reported.

## The Study

During September–December 2013, a total of 1,097 blood and spleen samples were collected from diseased or dead animals on Corsica; the samples were sent to the French National Reference Laboratory at Agence Nationale de Sécurité Sanitaire (Maisons-Alfort, France) to be tested for the presence of BTV. RNA was extracted from the samples and then amplified by using a real-time reverse transcription PCR (RT-qPCR). The ADIAVET BTV Real-time PCR Kit (bioMérieux, Saint Brieuc, France) was used for BTV group (i.e., *Orbivirus Bluetongue virus*) and serotype 1 detection. The LSI VetMAX European BTV Typing (1-2-4-6-8-9-11-16) Real-time PCR Kit (Life Technologies, Lissieu, France) was used for BTV-2, -4, -9, and -16 serotyping. A total of 531 samples from sheep, cattle, and goats had RT-qPCR results positive for BTV. All genotyped samples were positive only for BTV-1.

At the end of 2013, a compulsory vaccination program of domestic sheep, cattle, and goats was initiated by veterinary authorities in France. During the campaign, testing of ruminants with clinical signs of BTV was continued as part of a corresponding monitoring program.

During January–April 2014, we analyzed 436 samples from goats; 86 (19.7%) had positive BTV group RT-qPCR results. This method detects the BTV RNA genome segment 10. Of the 86 BTV-positive samples, 73 with a cycle threshold (C_t_) value of <35 were genotyped: 57 (78.0%) of the samples were classified as BTV-1, and 10 (13.7%) were classified as non–BTV-1, -2, -4, -9, -16, and -25. By using various primers (Table) with a conventional in-house RT-PCR that amplifies a region of the BTV RNA genome segment 2, we obtained an amplicon for each of the 10 samples that were negative for BTV-1, -2, -4, -9, -16, and -25 (segment 2 encodes viral protein 2 and determines the serotype). The animals from which these samples came were in 5 herds that were sampled in January and February 2014. One of the 10 amplified products (806 bp) was sequenced directly and compared with homologous sequences available in the GenBank database; Blast 2.2.28 (http://blast.ncbi.nlm.nih.gov/Blast.cgi) was used for the comparison. Because the genome sequence was similar to that of the BTV-25 segment 2, we selected different primers, and by gene walking, we obtained the full coding sequence of the gene.

**Table:** Primers and probe used to detect the BTV strain BTV-n in samples from goats, Corsica, France, 2014*

PCR type	Target	Probe and primer names	Primer sequence, 5′ → 3′	Nucleotide location
Conventional BTV RT-PCR	Segment 2	PF	YRWTTGATTTTGARAARGA	1549–1566
		PR	GAAYCGACCACTGCCTATG	2355–2337
Conventional RT-PCR	BTV-n segment 2	BTV-n F	CAGATCTGGTTTTACCGAG	1546–1564
		BTV-n R	ATGATCCATCGGACTTAACT	1949–1927
Real-time RT-PCR	BTV-n segment 2	BTV-n F3	TGGATCATGATGGTTATGAACACC	1942–1966
		BTV-n R3	CGCCTCTCCAATCTCACGTATT	2102–2081
		BTV-n	FAM-TGACTATGCGAGGTTGG-MGB	1987–2004

*BTV, bluetongue virus; RT, reverse transcription; PF, forward primer; PR, reverse primer.

BLASTN 2.2.29 (http://blast.ncbi.nlm.nih.gov/Blast.cgi) was used to align sequences: the virus showed highest identity with the BTV-25 and BTV-26 serotypes circulating in Switzerland and Kuwait, respectively (*6*,*7*) (Figure). The complete segment 2 sequence of this new virus from Corsica (termed BTV-n; GenBank accession no. KM200718) shared 73% nt and 75% aa identify with BTV-25 sequences and 65% nt and 60% aa identity with BTV-26 sequences.

**Figure:**
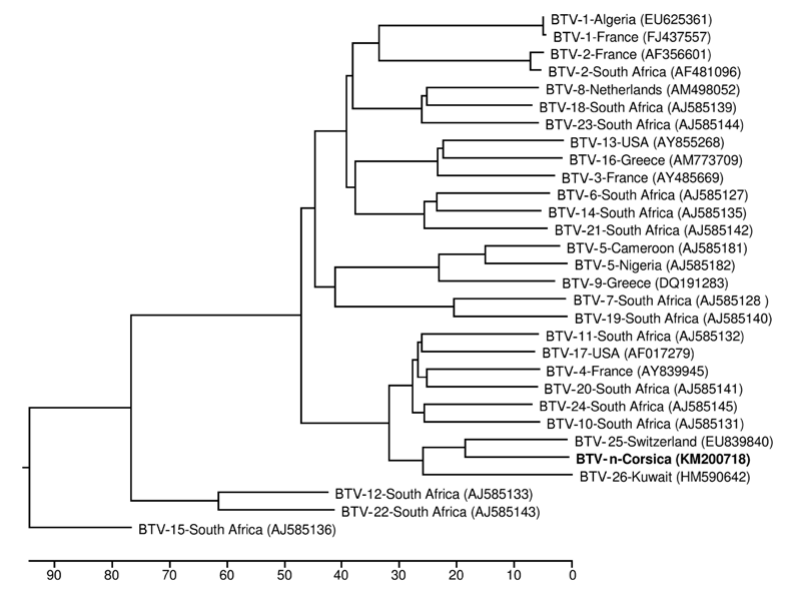
Phylogenetic tree of segment 2, showing relationships between BTV-n (boldface) and other BTV strains available in GenBank (accession nos. are shown in parentheses). Scale bar represents the percentage of nucleotide substitutions. BTV, bluetongue virus.

We developed a conventional in-house RT-PCR and an RT-qPCR that enabled specific detection of BTV-n by selection of BTV-n–specific primers and probe (Table). We could not detect BTV-n virus genome in any cattle or sheep samples tested in 2014, and we did not detect any co-infections with BTV-1 and BTV-n.

We attempted to isolate BTV-n from blood samples of the 10 BTV-n–positive goats; BTV-group C_t_ values for the samples ranged from 28.6 to 34.2. After many isolation attempts using embryonated chicken eggs as well as KC cells (a *Culicoides sonorensis*–derived cell line), Vero cells, and BHK (baby hamster kidney) cells, we isolated 1 virus strain by using BSR (a clone of BHK-21) cells. We used supernatants to conduct a BTV-n–specific RT-qPCR (primers shown in Table); the resulting C_t_ value of <17, indicated efficient virus replication. The virus was not neutralized by the reference antisera raised against BTV genotypes 1–24 and 26 or by an anti–BTV-25 serum (a titrated anti–BTV-25 serum is not available).

During May 2014, blood samples were collected from 56 goat bucks on a breeding farm located in the same area as the farms with the 4 previously sampled BTV-n–positive herds. Of the 56 sampled bucks, 51 were BTV-n positive by the BTV group–specific RT-qPCR (C_t_ value range 27.7–37.8). No buck was BTV-1 positive by the RT-qPCR, but 43 were positive by the BTV-n RT-qPCR; for the other 8 bucks, C_t_ values were beyond the positive range. Many of the bucks had not been vaccinated against BTV and showed no clinical signs of infection.

## Conclusions

We report the detection and identification of a new BTV, BTV-n, that is circulating among goats on Corsica. BLAST analysis of the BTV-n RNA segment 2 suggests that this virus most likely belongs to the BTV serogroup. However, full-genome segment 2 sequence identity between BTV-n and its closest BTV relative, BTV-25, was low (73.0% homologous); thus, BTV-n may be a novel serotype.

Some criteria for defining serotype/nucleotype have been defined by Maan et al. (*6*) and Hofmann et al. (*7*). Comparison of the segment 2 nucleotide sequences of the 26 BTV serotypes enabled definition of 12 nucleotypes; members of the same segment 2 nucleotype are characterized by at least 66.9% identity in their segment 2 nucleotide sequences (*7*). Our findings show that BTV-n shares 73.0% identity with the full genome segment 2 sequence of BTV-25. According to criteria defined by Maan et al. (*6*), BTV-n cannot be considered a new nucleotype and seems to belong to the BTV-25 nucleotype. Maan et al. (*8*) also showed that the differences in segment 2 sequences correspond to differences in serotypes. They reported overall interserotype variations in segment 2 of 29.0% (BTV-8 and BTV-18) to 59.0% (BTV-16 and BTV-22); the deduced amino acid sequence of VP2 varied from 22.4% (BTV-4 and BTV-20) to 73.0% (BTV-6 and BTV-22) (*8*). However, unlike BTV-25, BTV-n can grow in BSR cells (*7*,9).

Like BTV-25 (*9*) and BTV-26 strains, BTV-n has been detected in goats without clinical signs and symptoms of infection. All of the samples positive for BTV-n by RT-qPCR were from healthy bucks, and most BTV samples from clinically affected goats were negative for BTV group by RT-qPCR. Thus, it is also likely that BTV-n is not pathogenic for goats.

We suggest, on the basis of the following findings, that the newly detected BTV-n strain could be a new serotype: the differences between BTV-n sequences and other BTV sequences were as high as those described by Maan et al. (*8*); BTV-n is genetically diverse from other BTVs; BTV-n could not be amplified by the BTV-25–specific RT-PCR; anti–BTV-25 serum could not neutralize BTV-n; and, unlike BTV-25, BTV-n can be cultivated on BSR cells (*7*,*10*). No information is available about the origin of this new BTV strain; more in-depth investigation is needed to determine when and how the virus arrived in Corsica. Additional genome sequencing and experiments in various ruminant species are planned. These studies will help characterize this new BTV strain and evaluate the duration of viremia, humoral immune response, and pathogenicity of this serotype.
